# The Presence and Tissue Distribution of *Fusobacterium nucleatum* and *Porphyromonas gingivalis* in Oral Squamous Cell Carcinoma

**DOI:** 10.1155/ijod/5994390

**Published:** 2026-08-13

**Authors:** Juho-Matti Huhtala, Marwa Buaoud, Ahmed Musrati, Katariina Piiparinen, Auli Suominen, Caj Haglund, Jaana Hagström, Jaana Willberg

**Affiliations:** ^1^ Department of Oral Pathology and Oral Radiology, Institute of Dentistry, University of Turku, Turku, Finland, utu.fi; ^2^ Department of Oral and Maxillofacial Diseases, Turku University Hospital, Turku, Finland, vsshp.fi; ^3^ Department of Pathology, Libyan Centre for Dental Research, Zliten, Libya; ^4^ TYKS Laboratories, Department of Pathology, Turku University Hospital, Turku, Finland, vsshp.fi; ^5^ Department of Community Dentistry, Institute of Dentistry, University of Turku, Turku, Finland, utu.fi; ^6^ Translational Cancer Medicine Research Program, Faculty of Medicine, University of Helsinki, Helsinki, Finland, helsinki.fi; ^7^ Department of Surgery, University of Helsinki and Helsinki University Hospital, Helsinki, Finland, helsinki.fi

**Keywords:** immunohistochemistry, oral squamous cell carcinoma, periodontopathogens

## Abstract

**Purpose:**

The aim of our study was to investigate the presence of *Fusobacterium nucleatum* (FN) and *Porphyromonas gingivalis* (PG) in oral cancer samples, to possibly reveal their potential contribution to oral cancer.

**Methods:**

We examined a total of 48 histological specimens, of which 42 were squamous cell carcinomas, two verrucous carcinomas (VCs), and one adenocarcinoma (AC); two squamous cell papillomas and one verruca vulgaris (VV) served as controls. Samples were immunostained with specific antigens for FN and the enzyme Gingipain R1 representing PG, followed by visual assessment of staining intensity. The results were analyzed by cross tabulation to detect statistical significance between the groups.

**Results:**

FN and PG were commonly found simultaneously in tumor samples and more often in tumor and stroma compared to other tissues layers. In the epithelium, Gingipain was expressed more often than FN although often as co‐occured. The alveolar ridge had the lowest number of immunonegative samples for FN and Gingipain. Gingipain often showed higher staining intensity. We did not find correlations with patient age, sex, or anatomical tumor localization.

**Conclusions:**

Our findings suggest a possible role for FN and PG in the development and progression of oral squamous cell carcinoma (OSCC), supporting the hypothesis that periodontal pathogens may contribute to oral carcinogenesis. Co‐occurence of FN and PG further points to potential bacterial interactions influencing tumor behavior. Further studies with larger cohorts are warranted to explore these associations. The findings support the potential role of periodontitis as a risk factor for oral cancer.

## 1. Introduction

The role of infections in the development of cancer is increasingly recognized. It is estimated that infections contribute to up to 20% of all cancers [1]. Among head and neck cancers, for example, human papillomavirus (HPV) has been found to directly associate with the development of oropharyngeal carcinoma [2]. *Helicobacter pylori* has been linked to gastric cancer [3] and Merkel cell polyomavirus to Merkel cell carcinoma of the skin [4].

*Fusobacterium nucleatum* (FN) is a Gram‐negative bacterium of the oral microbiota, associated with gingivitis and periodontitis. Within dental plaque, FN is the second most common bacterium in the biofilm [5]. FN is known for its adhesion properties, which facilitate its attachment to host cells as well as to other bacterial species [6]. Elevated FN levels have been reported in head and neck cancer, and FN has been isolated from gastrointestinal cancers as well. In colorectal cancers (CRCs), elevated FN levels have been associated with poorer prognosis [7–10]. FN has several potential pathways through which it is thought to be able to influence the growth and development of malignancies, such as the growth of epithelial cells and the activation of tissue‐degrading enzymes like matrix metalloproteinases (MMPs) [11].

*Porphyromonas gingivalis* (PG) is another Gram‐negative oral bacterium strongly linked to periodontitis [12]. Elevated PG levels have been identified as a risk factor for oral squamous cell carcinoma (OSCC) [13, 14]. Like FN, PG has been found in gastrointestinal cancers, and in CRC, it is associated with poorer prognosis [15, 16]. PG produces several infection‐triggering virulence factors that are able to induce MMP activation, and it can promote cell division and angiogenesis, influences the regulation of normal apoptosis, facilitates invasion and metastasis, and thus helps carcinoma cells evade the immune response [17, 18].

Moreover, the coexistence of FN and PG enhances virulence in periodontitis and oral cancer models [19, 20]. However, research on their presence, individually or together, in human oral cancer remains limited. In this study, we examined human OSCC samples using immunohistochemistry and, for the first time in patient‐derived tumors, demonstrate both their individual presence and significant co‐occurence. Understanding the interactions between these microbes and oral cancer is essential to elucidate their potential role in tumor biology.

## 2. Materials and Methods

### 2.1. Sample Material

The samples (*n* = 48) were collected from the pathological archives of the Faculty of Dentistry, University of Benghazi, Libya, with the ethical permission of the IRB of the university. The data consisted of 42 OSCC samples, one adenocarcinoma (AC), two verrucous carcinomas (VCs), two squamous cell papillomas, and one verruca vulgaris (VV). Additionally, 10 benign oral mucosal biopsy samples were included in the study as control material. Control samples were collected from the archives of the Histology laboratory, Institute of Dentistry, University of Turku. The use of samples was authorized by Turku University and Turku University Hospital. All study samples were re‐evaluated by a specialist in oral pathology.

### 2.2. Immunohistochemistry

From formalin‐fixed paraffin‐embedded samples, 4–5 µm thick sections were collected on glass slides.

Slides were treated in a pretreatment module (Dako Agilent, Santa Clara, CA, USA) in retrieval solution (EnVision Flex target retrieval solution, high ph, DM828) and retrieval solution (EnVision Flex target retrieval solution, low ph, DM829) followed by blocking endogenous peroxidase (EnVision Flex peroxidase‐blocking reagent, SM801) according to the manufacturer`s instructions.

The immunostaining was performed by Autostainer 480 (Lab Vision Corp, Fremont, CA, USA) with primary antibodies for 1 h, followed by 30 min of incubation with the DAKO REAL Envision/HRP detection system and Rabbit/Mouse (EnVision) reagent.

The immunohistochemical staining for PG was performed with a polyclonal rabbit antibody against a specific proteinase of PG, Gingipain R1 (1:600, Biorbyt Ltd, Cambridge, UK). This antibody has previously been used to detect PG in the following studies [21, 22].

For FN, the staining was performed with a polyclonal rabbit antibody (1:100, Diatheva S.R.L, Cartoceto, ITA).

Slides were finally visualized using the EnVision Flex Substrate Working solution for Chromogen. Between each step, slides were washed with PBS‐0.04% Tween20. Slides were counterstained with Dako Meyer`s hematoxylin and mounted in a mounting medium (Aquamount, BDH Chemicals Ltd, Poole, UK). In each staining, gingival tissue was used as a positive control.

### 2.3. Immunohistochemical Scoring

**Figure 1:**
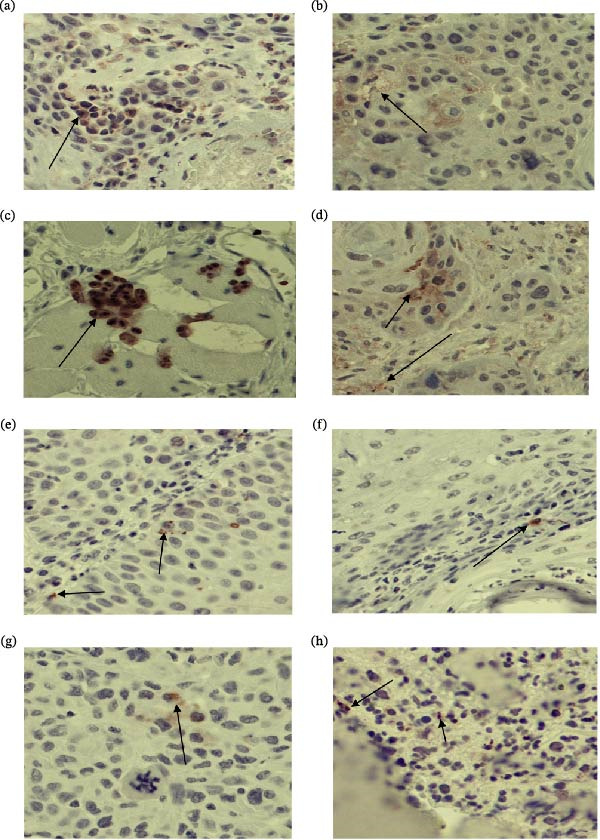
Immunohistochemical staining of oral cancer samples for *Porphyromonas gingivalis* (PG), performed with polyclonal rabbit antibody against Gingipain R1 (a–d). Gingipain staining detected in stromal (a) and in tumor cells (b). Strong Gingipain staining detected in neutrophils (c) and in stromal and tumor cells (d). Immunohistochemical staining of oral cancer samples for *Fusobacterium nucleatum* (FN), photographs (e–h). Mild positivity for FN in tumor (e) and stromal cells (g). Mild stromal FN positivity detected in inflammatory cells (f, h). All photographs are taken at 400x magnification.

Gingipain and FN immunohistochemical positivities were scorjed by two researchers (Juho‐Matti Huhtala and Jaana Willberg) separately, blinded to clinical data. Any discordance in scoring was resolved by reassessment in order to achieve consensus. Scoring was based on the intensity of positivity: none = 0, mild = 1, moderate = 2, or strong = 3. The tissue layers that were scored were epithelium, tumor, stroma, muscle fibers, capillary wall epithelium, and salivary gland (Figure 1).

**Figure 2:**
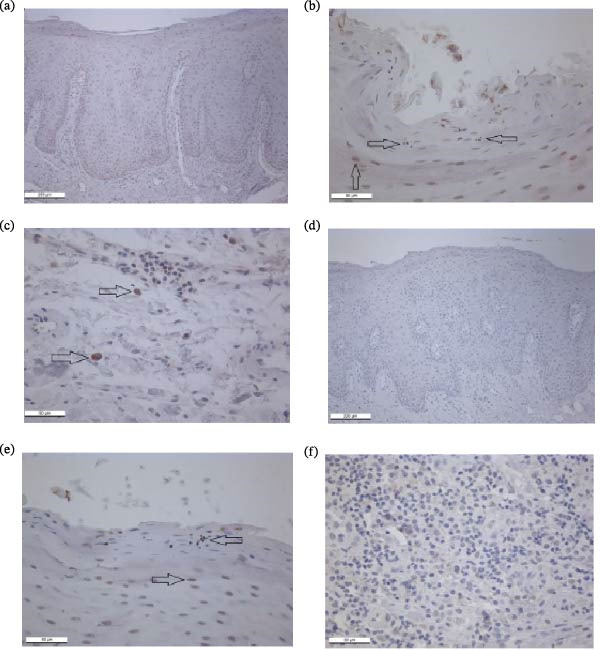
Immunohistochemical staining of control samples including benign oral mucosal samples for PG (a–c) and FN (d–f). Gingipain and FN staining were both negative in the epithelium and connective tissue stroma of normal oral mucosa (a, d). Gingipain and FN positivity was detected in some microbial colonies on the surface and in the upper epithelial layers of inflammatory lesions (b, e). Faint positive staining of Gingipain was detected in few inflammatory cells among connective tissue (c) whereas FN was negative in inflammatory cells (f).

Immunostaining profile of Gingipain and FN in control samples of benign oral mucosal tissue was described (Figure 2). Images of immunostainings were taken with a digital camera (Leica DFC320, Leica Microsystems) and imaging software (Leica Application Suite version 4.1.0, Leica Micrsosystems) connected to a light microscope (Leitz Aristoplan, Leica Microsystems, Weztlar, Germany).

### 2.4. Statistical Analyses

The difference between the scoring groups was analyzed using cross‐tabulation.

Statistical analysis was performed using SPSS version 29.0 (IBM SPSS Statistics, IBM Corporation, New York, NY, USA).

Associations between categorical variables were evaluated by the Fisher–Freeman–Halton exact test and Pearson chi‐square test. The Fisher–Freeman–Halton test was used if the assumptions of the Pearson chi‐square test were not met. Kruskal–Wallis test was used for the comparison of age between intensity groups, as continuous age was not normally distributed. The scorings between Gingipain and FN groups were compared with the Wilcoxon rank‐sum test. Statistical significance was considered at *p* < 0.05.

## 3. Results

### 3.1. FN and Gingipain Staining Intensities Compared to Age and Sex

Patient age information was available for 43 FN‐ and 44 Gingipain‐stained samples. For both groups, the mean age was 58 years, and the median age was 60 years, with an overall age range of 12–93 years for all cases.

No statistically significant difference in age was found by the Kruskal–Wallis test between FN or Gingipain (FN *p* = 0.462, Gingipain *p* = 0.452).

Patient gender information was available for 46 samples stained for FN and for 47 samples stained for Gingipain. No statistical significance was found between staining intensities and gender (FN *p* = 0.283, Gingipain *p* = 0.581).

### 3.2. FN and Gingipain Staining Intensities Compared to Diagnosis (Tables 1, 2)

The staining intensity of FN was moderate or strong only in OSCC samples. Staining intensity was mild in most cases, 54.4% of all FN‐positive samples. Negative samples totaled 17.4% of all samples, of which 25% (*n* = 2) were non‐OSCC samples.

**Table 1:** *Fusobacterium nucleatum* (FN) staining intensities compared to diagnosis.

FN intensity	OSCC	AC	VC	VV	Papilloma	Total (*n*)
Negative	6	0	0	1	1	8
	13.0%	0.0%	0.0%	2.2%	2.2%	
+	21	1	2	0	1	25
	45.7%	2.2%	4.3%	0.0%	2.2%	
++	8	0	0	0	0	8
	17.4%	0.0%	0.0%	0.0%	0.0%	
+++	5	0	0	0	0	5
	10.8%	0.0%	0.0%	0.0%	0.0%	

*Note*: Papilloma, squamous cell papilloma.

Abbreviations: AC, adenocarcinoma; OSCC, oral squamous cell carcinoma; VC, verrucous carcinoma; VV, verruca vulgaris.

**Table 2:** Gingipain staining intensities compared to diagnosis.

Gingipain intensity	OSCC	AC	VC	VV	Papilloma	Total (*n*)
+	24	0	1	1	1	27
	51.1%	0.0%	2.1%	2.1%	2.1%	
++	12	0	1	0	1	14
	25.5%	0.0%	2.1%	0.0%	2.1%	
+++	5	1	0	0	0	6
	10.6%	2.1%	0.0%	0.0%	0.0%	

*Note*: Papilloma, squamous cell papilloma.

Abbreviations: AC, adenocarcinoma; OSCC, oral squamous cell carcinoma; VC, verrucous carcinoma; VV, verruca vulgaris.

None of the samples were negative for Gingipain. The staining intensity was strong in 12.7% of samples; of these, 83.3% (*n* = 5) were OSCC samples. AC showed a strong staining intensity. A mild intensity was seen in 57.4% of all samples.

The staining intensity of both FN and Gingipain was stronger in OSCC samples compared to samples with other diagnoses.

### 3.3. FN and Gingipain Staining Intensities in Relation to Tumor Location

Staining intensities showed no significant association with tumor location.

### 3.4. FN and Gingipain Staining Intensities Compared (Table 3)

There were statistical differences between FN intensity and Gingipain intensity (*p* = 0.043). Of the studied samples, 40% (*n* = 18) showed similar intensities for FN and Gingipain. In 15.6% (*n* = 7) of samples, FN showed stronger intensity than Gingipain, and in 44.4% (*n* = 20) of samples, Gingipain intensity was stronger than FN intensity. Co‐occurence of FN and Gingipain was found in 83% (*n* = 38) of samples.

**Table 3:** *Fusobacterium nucleatum* and Gingipain staining intensities compared.

Intensity	Total (*n*)	Neg	+	++	+++	Wilcoxon rank‐sum test
*F. nucleatum*	46	8	25	8	5	0.043
Gingipain	47	0	27	14	6	

### 3.5. FN and Gingipain Immunoexpression in OSCC Tissue Layers (Tables 4, 5)

For FN staining, there were a total of 48 samples, of which 46 were included in the statistical analysis. In two samples, the staining intensity could not be measured because of the poor quality of the histological sample.

**Table 4:** *Fusobacterium nucleatum* staining intensities compared to tissue layers.

*F. nucleatum* intensity	Total (*n*)	Negative	+	++	+++	Pearson chi‐square
Tissue layer						
Epithelium	46	37	5	2	2	<0.001
		80.4%	10.9%	4.3%	4.3%	
Stroma	46	17	17	7	5	0.013
		36.9%	36.9%	15.2%	10.8%	
Tumor	46	18	17	6	5	0.006
		39.1%	37.0%	13.0%	10.9%	
Capillary	46	41	5	0	0	<0.001
		89.1%	10.9%	0.0%	0.0%	
Salivary gland	46	44	1	0	1	<0.001
		95.6%	2.1%	0.0%	2.1%	
Muscle fibers	46	36	7	2	1	<0.001
		78.2%	15.2%	4.3%	2.1%	

**Table 5:** Gingipain staining intensities compared to tissue layers.

Gingipain intensity	Total (*n*)	Negative	+	++	+++	Pearson chi‐square
Tissue layer						
Epithelium	47	16	13	13	5	0.128
		34.0%	27.6%	27.6%	10.6%	
Stroma	47	2	25	14	6	<0.001
		4.2%	53.1%	29.7%	12.7%	
Tumor	47	6	23	12	6	<0.001
		12.8%	48.9%	25.5%	12.8%	
Capillary	47	46	1	0	0	<0.001
		97.8%	2.1%	0.0%	0.0%	
Salivary gland	47	43	1	2	1	<0.001
		91.4%	2.1%	4.2%	2.1%	
Muscle fibers	47	40	5	1	1	<0.001
		85.1%	10.6%	2.1%	2.1%	

For FN, the highest positivity was detected in tumor and stroma. In tumor cells, 62.9% of cases had FN positivity, while 60.8% had stromal positivity. For FN, the epithelium showed positivity in 19.5% of cases. In small capillaries, FN was found in 10.9% of samples and in salivary glands only in 4.2% of samples. FN positivity in muscle fibers was detected in 21.6% of the samples.

Non‐OSCC samples were either negative or showed mild intensity for FN.

In tumor and stroma FN enrichment was statistically significant compared to epithelium (*p* < 0.001), capillaries (*p* < 0.001), muscle fibers (*p* < 0.001), and salivary glands (*p* < 0.001). No statistically significant difference was found between tumor and stroma.

For Gingipain, we had 48 samples, of which 47 were included in the analysis. One sample could not be reliably evaluated for staining intensity; it was a different sample than the one for FN. Gingipain showed the highest positivity also in tumor and stroma. In tumor cells, the positivity for Gingipain was detected in 87.2% of cases. In stroma, Gingipain positivity was found in 95.7% of cases. In epithelium, Gingipain showed positivity in 65.9% of cases. Gingipain was detected in small capillaries in 2.12%, in salivary glands in 8.5%, and in muscle fibers in 14.8% of cases.

Non‐OSCC was either mild or moderate intensity for Gingipain.

Statistically significance was found for Gingipain expression in tumor compared to epithelium (*p* = 0.018), capillaries (*p* < 0.001), salivary glands (*p* < 0.001), and muscle fibers (*p* < 0.001). No statistical significance was found between tumor and stroma. Stromal positivity was statistically significant compared to epithelium (*p* < 0.001), capillaries (*p* < 0.001), salivary glands (*p* < 0.001), and muscle fibers (*p* < 0.001).

### 3.6. FN and Gingipain Staining in Control Samples

FN and Gingipain staining were negative in the epithelium and connective tissue of healthy oral mucosa (Figure 2). In the areas of mucosal inflammation, FN and Gingipain staining was positive within microbial colonies locating on the surface and in the upper layers of the epithelium. In the same samples, faint FN and Gingipain positivity was detected in upper epithelial cells. FN was negative in inflammatory cells in the connective tissue and among muscle tissue. Gingipain was positive in few inflammatory cells in the connective tissue in seven samples, and single immunopositive inflammatory cells were detected among the muscle tissue in four samples.

## 4. Discussion

The present study aimed to evaluate the presence of FN and PG, either individually or in combination, in OSCC. Consistent with this objective, FN was detected in 83% of oral cancer samples, while Gingipain was present in all samples (100%). FN and Gingipain were not detected in control samples of normal oral mucosa, and in the presence of mucosal inflammation, they were located in the upper layers of the epithelium and Gingipain additionally in few inflammatory cells. Although their presence in tumor samples was not significantly associated with age, gender, or tumor location, these findings suggest that FN and Gingipain may act as independent risk factors in oral cancer development. This observation aligns with recent studies reporting a high prevalence of these bacteria in malignant tissues, further supporting their potential pathogenic role in the causation of oral cancer [7, 10]. It should be noted that Gingipain is not directly related to the level of PG, but it provides an indication of bacterial presence in the tissue.

Despite the lack of a significant association between FN and Gingipain levels and tumor location, samples from the alveolar ridge had the fewest negative cases relative to the total number of samples. This finding aligns with Katz et al. [23] who reported high PG concentrations in gingival carcinomas and could support the hypothesis of a possible influence of periodontal diseases on carcinoma formation.

In OSCC samples, FN expression was detected in both tumor cells and the surrounding stroma, whereas Gingipain was predominantly localized in the epithelium. This difference may be explained by the pathogenic mechanisms of Gingipain, the major virulence factor of PG, whose primary function is to attach to epithelial cells and promote pathogen invasion of tissues [24]. The localization of these bacteria suggests that FN and Gingipain, through their proteolytic activity, may interact with both malignant epithelial cells and the tumor microenvironment, potentially contributing to tumor progression, invasion, and local immune modulation. Supporting this notion, studies on *Treponema denticola*, another periodontal pathogen, have shown that invasion depth correlates positively with tumor size of tongue cancer and that this bacterium is particularly expressed in aggressive disease forms [25]. Taken together, these findings underscore the potential role of periodontopathogens, including FN and PG, in oral cancer development and progression.

Interestingly, we found stronger FN and Gingipain staining in OSCC samples compared with non‐OSCC samples. This finding aligns with previous reports showing a higher abundance of FN and PG in malignant compared to benign oral tumors, suggesting a possible role in the tumor microenvironment [26, 27].

In some samples of our study, FN and Gingipain positivity was present in the muscle layer and around the capillaries. This leads us to speculate that FN and PG might be involved in the metastatic phenomenon. The interesting finding that FN was found in muscle tissue and blood vessels as well needs further research to be done, although we know that this bacteria is capable to invade surrounding tissues [28]. Previous studies have found FN prevalence predicting metastasis in CRC patients, and elevated levels increase the risk of metastasis [29]. PG on the other hand can activate MMPs that, upon activation, stimulate OSCC cell proliferation, invasion, and metastasis [30, 31].

Although the expression intensities of FN and Gingipain varied significantly, we observed that FN was strongly expressed, while Gingipain intensity was comparatively lower. This pattern supports the role of FN as an intermediate colonizer [32], enabling the attachment and persistence of late‐colonizing bacteria, such as PG [33]. Nevertheless, their co‐occurence in 83% of OSCC samples underscores the importance of microbial interactions rather than the influence of individual species alone. This finding is consistent with Song et al. [20], who reported that the coexistence of FN and PG synergistically enhanced tumor‐promoting effects in OSCC cell line models. Similarly, Gallimidi et al. demonstrated in a mouse model that FN/PG‐infected tongue tumors were 2.5 times larger and significantly more invasive than uninfected controls, further supporting the concept of co‐operative microbial interactions within the tumor microenvironment [34].

The limitations of this study include the relatively small sample size and the subjective nature of immunohistochemical scoring. Furthermore, the absence of previous studies employing the same methodology and sample type limits the direct comparison and contextualization of our findings.

To conclude, FN and PG may influence the development of OSCC, especially when they co‐occur in the same area. The findings of our study support the contribution of periodontitis as a possible risk factor for oral cancer. We can speculate that as FN is an intermediate colonizer, it may help PG adhesion and invasion, and together, they may take part in cancer development.

## Funding

This work was supported by the Turku University Hospital Research Foundation and Helsinki University Hospital funding (Grant Y780024004). Open access publishing facilitated by Turun yliopisto, as part of the Wiley ‐ FinELib agreement.

## Ethics Statement

University of Benghazi Libya oral pathological archives with the ethical permission of the university. The use of control samples was authorized by Turku University and Turku University Hospital.

## Conflicts of Interest

The authors declare no conflicts of interest.

## Data Availability

The data that support the findings of this study are available from the corresponding author upon reasonable request.
